# Demographic comparison of sweetpotato weevil reared on a major host, *Ipomoea batatas,* and an alternative host, *I. triloba*

**DOI:** 10.1038/srep11871

**Published:** 2015-07-09

**Authors:** Gadi V. P. Reddy, Hisn Chi

**Affiliations:** 1 Western Triangle Agricultural Research Center, Montana State University, 9546 Old Shelby Rd., P. O. Box 656, Conrad, MT 59425, USA; 2 Laboratory of Theoretical and Applied Ecology, Department of Entomology, National Chung Hsing University, Taichung 402, Taiwan, Republic of China

## Abstract

In this study, we collected life table data for the sweetpotato weevil, *Cylas formicarius,* grown on *Ipomoea batatas* and *Ipomoea triloba*, and analyzed them using an age-stage, two-sex life table. We also demonstrated the growth potential of *C. formicarius* on these two host plants by using population projection. These data will be useful to the growers to the selection or eradication of host plants in an integrated control strategy for *C. formicarius* for the entire area of the targeted areas. We found that *C. formicarius* developed faster on *I. batatas* than on *I. triloba*. The developmental times of the larval and pupal stages on *I. batatas* than on *I. triloba* were 37.01 and 8.3 days. The adult females emerged before and began to produce eggs at 42 days earlier when reared on *I. batatas*. The fecundity of females was 90.0 eggs on *I. batatas* significantly higher than the mean fecundity of 68.5 eggs on *I. triloba*. Although this insect has a higher intrinsic rate of increase on *I. batatas*, the study indicated that *C. formicarius* can successfully survive and reproduce on both host plants.

A life table is a convenient and comprehensive method for summarizing the survival and reproductive potential of a population. Using a life table, one can compare the growth potential of an insect on different host plants, under different environmental conditions^1^,^2^,^3^. Because the age-stage, two-sex life table can describe stage differentiation and include both sexes, it can precisely reveal the actual life history of the insect species and life table have been widely applied in the study of various ecological aspects of interest in relation to insect pests and their natural enemies^4^. Understanding the demography of an insect under variable conditions is the cornerstone for developing an environmentally friendly pest management strategy.

The sweetpotato weevil, *Cylas formicarius* (F.) (Coleoptera: Brentidae), is the most serious pest of sweet potato (*Ipomoea batatas* [L.] Lam., Convolvulaceae)^5^ both in the field and in storage^6^. In the Marianas Islands, *Ipomoea triloba* L. (Convolvulaceae) (littlebell, or Aiea morning glory) is widespread and serves as an alternative host for *C. formicarius*. This vine is reported to grow around sweet potato fields and has been recorded as an alternate host of another pest of sweet potato *Euscepes postfasciatus* (Fairmaire) (Coleoptera: Brentidae)^7^. In addition to *I. batatas*, the major host plant of *C. formicarius*^5^, at least 49 other members of the Convolvulaceae have been recorded as hosts for *C. formicarius,* which has been recorded feeding on seven genera in six tribes within this plant family^8^,^9^. In Guam and other Micronesian Islands, *I. triloba* is widespread and serves as an alternative host for *C. formicarius*^10^. Because of the cryptic nature of the larvae and the nocturnal activity of the *C. formicarius* adults, it is difficult to control this pest using chemicals^11^. In addition, the understanding the life history of *C. formicarius* will help and makes the pest easiest to control with long-residual pesticides that are now out of favor and often unavailable^5^.

To systematically understand the role of main and alternative hosts in the population explosion of *C. formicarius,* information on its demography, including development, survival, and fecundity on each host plant, is needed. In this study, we measured these life table parameters of *C. formicarius* on *I. batatas* and *I. triloba*, and analyzed them using an age-stage, two-sex life table. Furthermore, we estimated the potential rate of growth of *C. formicarius* on each of these two host plants using a population projection. If *C. formicarius* can survive on *I. triloba*, growers have to focus in controlling pest incidence on the alternative host as well. These data will be useful in the selection or eradication of host plants for an integrated control strategy for *C. formicarius*.

## Materials and methods

The experiments were conducted from January 2013 to March 2014 in a shade house at the Western Pacific Quarantine Biocontrol Laboratory of the University of Guam (13.43 °N, 144.80 °E, and 54.3 m).

### Study area

The shade house was 100 m^2^ in area; the walls and roof were constructed of shade cloth. Mean temperature in the shade house was 31 °C (range 29.5–32.5 °C), mean relative humidity was 78% (range 76–80%), and the natural photoperiod was 14–16:10-8 (L:D) h.

### Plants

Sweet potato (*I. batatas*) and Aiea morning-glory (*I. triloba*) cuttings (ca. 30 cm) were planted in individual pots (20 cm diameter × 30 cm deep) filled with disease- and weed-free Miracle Grow potting soil. One hundred and fifty such pots were planted with each of the two plant species for use in the experiments. Fertilizer in the form of Nitrogen, Phosphorus, Potassium, and Sulfur was applied at the actual time of planting according to published recommendations^12^.

### Insects

Pheromone lures consisting of rubber septa loaded with *Z*3-dodecenyl-*E*2-butenoate, sealed in an impermeable bag for shipping and storage, were obtained from Chem Tica Internacional S.A. (San José, Costa Rica)^13^. Pherocon unitraps (Trécé Incorporated, Adair, Oklahoma, USA) baited with these lures were used to trap adult *C. formicarius* in sweet potato fields in Latte Heights (Guam, USA) during 2010^14^. The trapped adults were taken to the laboratory, placed in batches in collapsible cages (12 × 10 × 10 cm), fed leaves and pieces of the sweet potato, and maintained at 22 ± 2 °C, 70–80% relative humidity and a 16:8 h L:D photoperiod^15^. In this colony, 5-6 generations were completed before the offspring were used for these experiments. For all experiments, 3-4 week-old adults were obtained from these laboratory colonies^16^.

### Experiments

Each pupae were taken out of the insect colony and placed in an individual test tube. Upon emergence, fifty pairs of adults for each plant species consisting of virgin males and females were taken; each female and male was placed on the both potted plants. Enlarged stalks and woody plants of (comparable sizes of *I. batatas* and *I. triloba*) were chosen whenever possible, as females prefer to deposit eggs on these plants. The plants were covered with collapsible rearing cages (30 × 30 × 60 cm) to prevent escape. Eggs were deposited singly, in cavities or cells formed by the adult in roots and vines of the host plant^5^. The egg is creamy white, broadly oval, and narrowed at the attached end. The egg becomes darker just before hatching, and the dark head of the larva becomes noticeable^17^. Data were taken daily on the number of eggs laid, the number hatched and the duration of each developmental stage of *C. formicarius* on the two host plants. For the life table studies a total of 150 eggs, all laid within 24 h, were collected from the 50 pairs for both *I. batatas* and *I. triloba.* The eggs were checked daily until they hatched and then checked the larvae daily for survival. Since immature stages of the *C. formicarius* grow inside host plant, the stems have been taken out of the cages observed the immatures’ growth and survival. Daily counts of surviving larvae at each stage as well as number of eggs laid by each female were recorded.

### Statistical analysis

Raw data of daily development and reproduction of each individual were analyzed according to the age-stage, two-sex life table theory^18^ as described by Chi^2^. The population parameters calculated were the age-stage survival rate (*s*_*xj*_, the probability that a newly laid egg will survive to age *x* and stage *j*), the age-specific fecundity of female (*f*_*xj*_, the mean fecundity of females at age *x*), the age-specific survival rate (*l*_*x*_, the probability of a newly laid egg survives to age *x*), and the age-specific fecundity (*m*_*x*_, the mean fecundity of individuals at age *x*). In the age-stage, two-sex life table, the *l*_*x*_ and *m*_*x*_ are calculated as


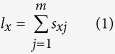



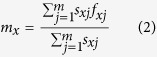


where *m* is the number of stages.

The net reproductive rate (*R*_0_) is calculated as


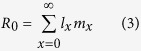


The intrinsic rate of increase (*r*) is estimated by using iterative bisection method from


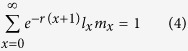


with age *x* indexed from 0^19^. The finite rate of increase (*λ*) is calculated as *λ* = *e*^*r*^. The mean generation time is defined as the length of time that a population needs to increase to *R*_0_-fold of its size (i.e., *e*^*rT*^ = *R*_0_ or *λ*^*T*^ = *R*_0_) at the stable age-stage distribution, and is calculated as


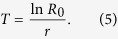


The life expectancy (*e*_*xj*_) of individuals at age *x* and stage *j* is calculated according to Chi and Su^4^, while the reproductive value (*v*_*xj*_) is calculated according to Tuan *et al*^20^. The means and standard errors of the life table parameters were estimated by using the bootstrap procedure^21^ with bootstrap number *m* = 40,000 to ensure more precise estimates. TWOSEX-MSChart Visual BASIC (version 6, service pack 6) for Windows (available at http://140.120.197.173/Ecology/ (Chung Hsing University) and http://nhsbig.inhs.uiuc.edu.tw/www/chi.html (Illinois Natural History Survey) were used to analyse our age-stage, two-sex life table data (Chi, H. TWOSEX-MSChart: a computer program for the age-stage, two-sex life table analysis. (http://140.20.197.173/Ecology/Download/Twosex-MSChart.rar, 2013)

The paired bootstrap test^21^,^22^ was used to compare the differences in developmental time, adult longevity, adult preoviposition period (APOP), total preoviposition period (TPOP), oviposition days, and fecundity between treatments. The population parameters (*r*, λ, *R*_0_, and *T*) between two treatments were also compared by using the paired bootstrap test based on the confidence interval of differences^21^,^22^.

#### Population projection

Survival rate and fecundity data were used to project population growth according to Chi and Liu^18^ and Chi^23^. The computer program TIMING-MSChart (Chi, H. TWOSEX-MSChart: a computer program for age-stage, two-sex life table analysis, http://140.120.197.173/Ecology/, 2014) used in this projection is also available at the above-mentioned web sites.

## Results

Out of 150 eggs (*N*) used at the beginning of life table study on *I. batatas*, 149 eggs hatched and 146 larvae successfully developed to adult stage (Table 1). We found that larvae and pupae of *C. formicarius* developed faster on *I. batatas* than on *I. triloba*. The developmental times of the larval and pupal stages were 37.0 and 8.3 days, respectively, when reared on *I. batatas*, which were significantly shorter than those observed on *I. triloba* (54.9 and 16.9 days, respectively) (Table 1). Adult females emerged earlier and began to produce eggs at 42 days earlier when reared on *I. batatas*. There was no difference between the adult preoviposition periods (APOP) reared on *I. batatas* and *I. triloba* (*P* = 0.5234). The mean of total preoviposition period (TPOP) on *I. batatas* was 50.1 d. When reared on *I. triloba*, the first female adult emerged much later (day 60) and the mean TPOP was 77.2 d, significantly later than on *I. batatas*. The mean fecundity (*F*) of females was 90.9 eggs on *I. batatas*, significantly higher than the mean fecundity of 68.57 eggs on *I. triloba* (Table 1). Out of 150 eggs (*N*) used at the beginning of life table study on *I. batatas*, 90 individuals emerged as female adults (*N*_*f*_); meanwhile, the values of *F*, *N*_*f*_, and *N* on *I. triloba* were 68.57, 83, and 150, respectively.

**Figure 1:**
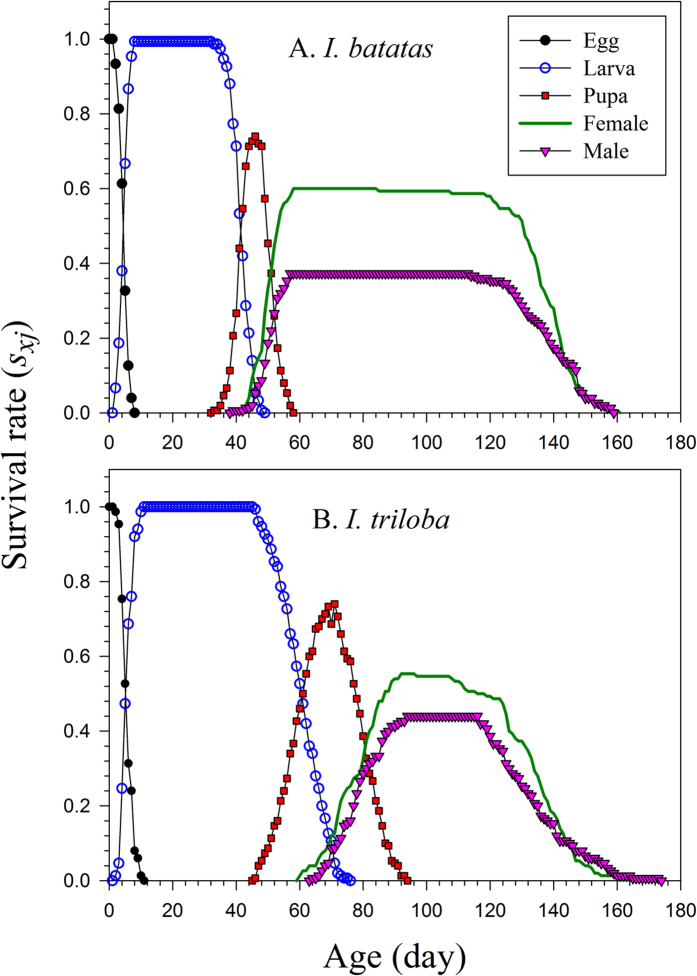
Survival rate to different developmental stages of *Cylas formicarius* on sweet potato *Ipomoea batatas* (A) and morning glory *Ipomoea triloba* (B).

**Figure 2:**
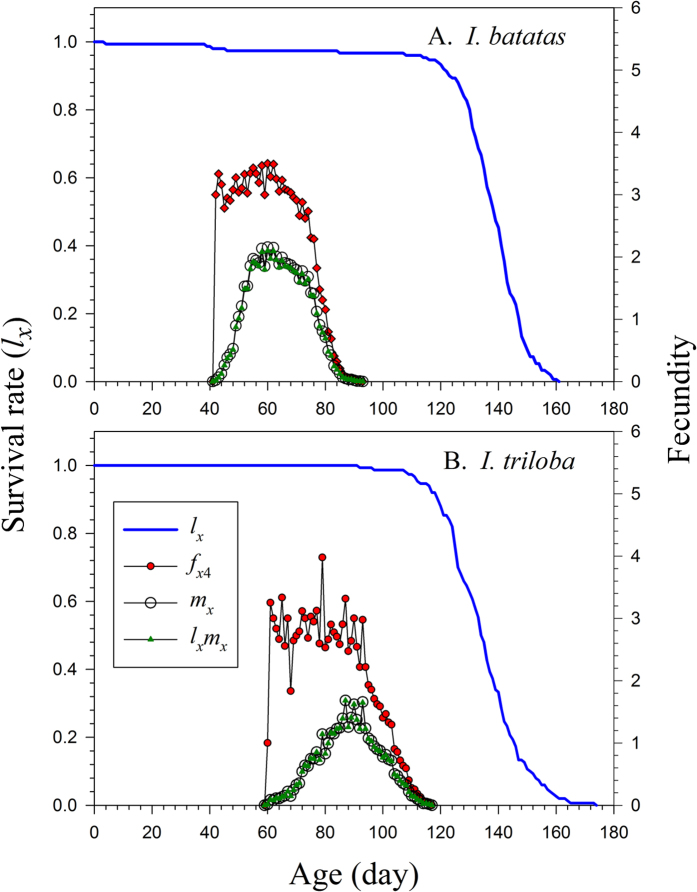
Age-specific survival rate (*l*_*x*_) versus age of *Cylas formicarius* on sweet potato *Ipomoea batatas* (A) and morning glory *Ipomoea triloba* (B).

The faster development on *I. batatas* could also be observed in the age-stage survival rate (*s*_*xj*_). The curves of females and males emerged at 42 and 39 d, respectively, for *I. batatas* (Fig. 1A); in contrast, female and male adults appeared at 60 and 64 d, respectively, on *I. triloba* (Fig. 1B). The female age-specific fecundity (*f*_*xj*_) and age-specific fecundity (*m*_*x*_) on *I. batatas* not only began much earlier (at 42 d) than that on *I. triloba* (at 60 d) (Fig. 2), but were also significantly higher than for the latter.

The intrinsic rate of increase (*r*), the finite rate (λ) and the net reproductive rate (*R*_0_) of the sweetpotato weevil reared on *I. batatas* were 0.0622 d^−1^, 1.0663 d^−1^, and 54.55 eggs, respectively, all significantly higher (*P *= 0.0001) than the values obtained on *I. triloba* (0.0418 d^−1^, 1.0426 d^−1^, and 37.94 eggs, respectively) (Table 2). On the other hand, the mean generation time (*T*) obtained on *I. batatas* (62.26 d) was shorter than that on *I. triloba* (87.07 d).

**Figure 3:**
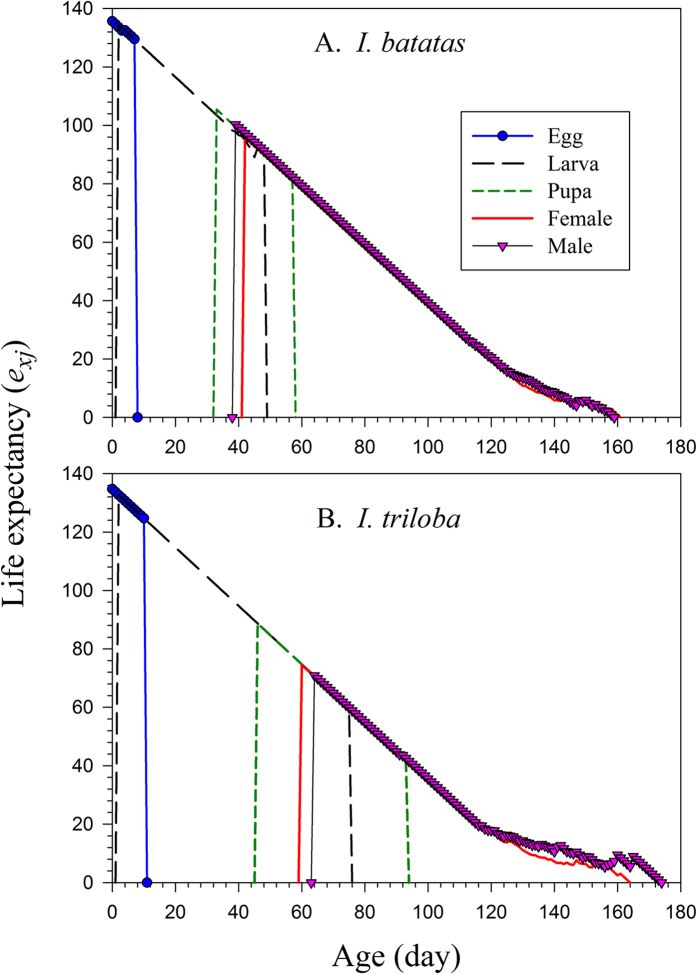
Life expectancy of *Cylas formicarius* on sweet potato *Ipomoea batatas* (A) and morning glory *Ipomoea triloba* (B).

**Figure 4:**
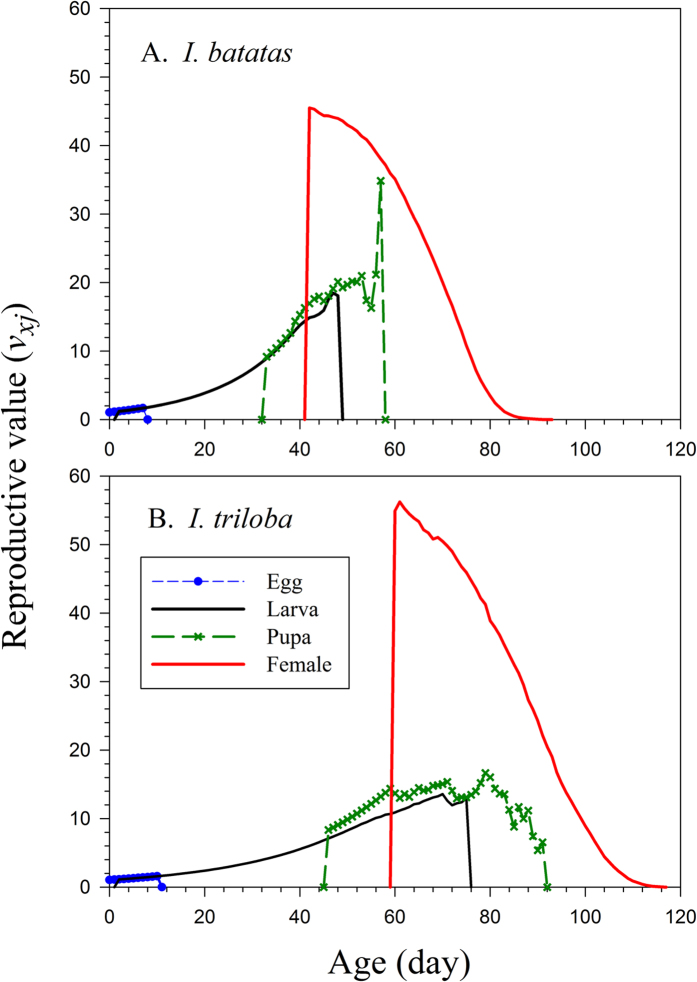
Reproductive value of *Cylas formicarius* on sweet potato *Ipomoea batatas* (A) and morning glory *Ipomoea triloba* (B).

The longevity of the *C. formcarius* at age zero (*e*_01_) was 135.63 d on *I. batatas,* which did not differ significantly from the longevity of 134.68 d on *I. triloba* (Fig. 3). These were exactly the mean longevity of all individuals used in the life table study. At age zero, the reproductive values (*v*_01_) were exactly the same as the finite rates on both host plants, i.e., 1.0663 d^−1^ on *I. batatas* and 1.0426 d^−1^ on *I. triloba* (Fig. 4). The value of *v*_*xj*_ on *I. batatas* jumped to 45.5 d^−1^ at 42 d when female adults emerged; when reared on *I. triloba*, the *v*_*xj*_ value jumped to 54.93 d^−1^ when female emerged later at 60 d.

**Figure 5:**
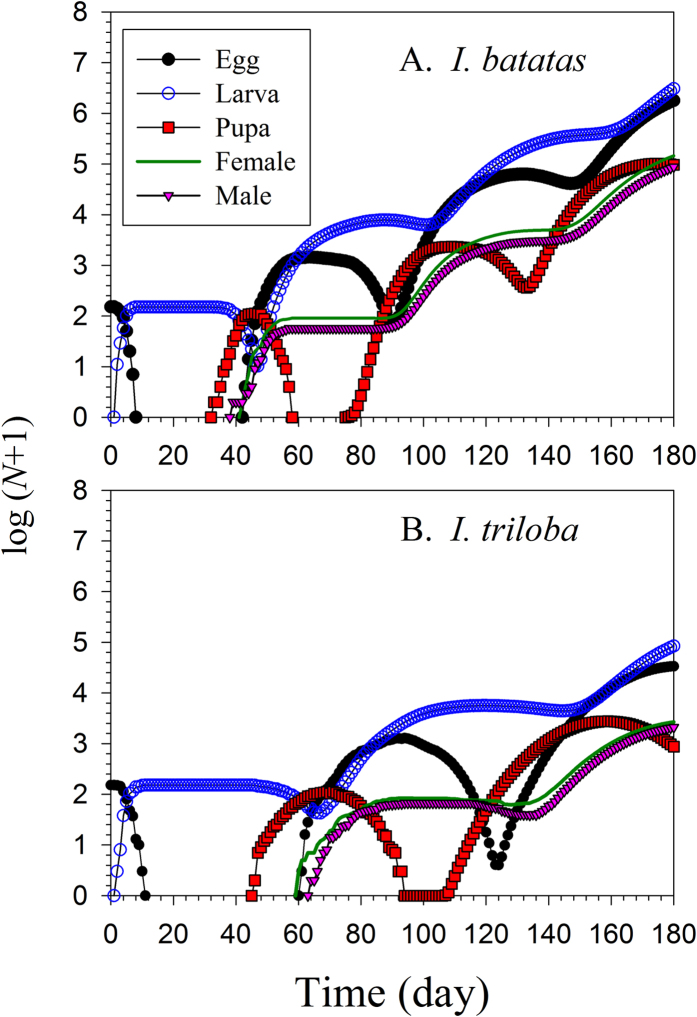
Population projection of *Cylas formicarius* on sweet potato *Ipomoea batatas* (A) and morning glory *Ipomoea triloba* (B). Population projection started with 150 eggs of *C. formicarius.*

The population projection suggested that *C. formicarius* will grow much faster on *I. batatas* than on *I. triloba* (Fig. 5). Beginning with 150 eggs, the population would undergo four generations, and the total population would exceed five million on *I. batatas* after 180 d, while the weevils would go through only three generation on *I. troloba*, for a final size of approximately 124,000.

## Discussion

The main host of *C. formicarius* and all species of sweetpotato weevil (*Euscepes postfasciatus* (Fairmaire), *Daealus tuberosus* (Zimmerman) and *Cylas puncticollis* (Boheman) is the sweet potato^24^,^10^,^25^. Another species, *Cylas brunneus* (Fabricius) has found only on sweet potatoes. Carrot (*Daucus carota* (Hoffm.) Schübl. & G. Martens), radish (*Raphanus sativus* L.) and morning glory (*I. triloba*) are known to serve as additional hosts for *C. formicarius*^24^. Since *I. triloba* is widespread in the Pacific Islands and other regions of the world^15^, it is one of the main alternative hosts for *C. formicarius*.

Because the age-stage, two-sex life table takes the variable developmental rate among individuals into consideration, the overlapping between stages can be observed in the curves of *s*_*xj*_. However, if the same data were analyzed using the traditional female age-specific life table, such as Lewis-Leslie matrix^26^,^27^,^28^,^29^, the stage differentiation would not be revealed. The age-specific survival rate (*l*_*x*_) (Fig. 2) is the simplified version of the age-stage survival rate (Fig. 1). Although the stage differentiation and overlapping could not be observed in the *l*_*x*_ curve, it is nevertheless constructed by using the age-stage, two-sex life table, and could correctly describe the change of the survival rate with age. Huang and Chi^30^ demonstrated that an erroneous *l*_*x*_ curve would be obtained if the traditional female age-specific were used.

Chi^2^ proved mathematically the relationship among *F*, *N*_*f*_, *N*, and *R*_0_ as *R*_0_ = *F* × (*N*_*f*_ /*N*). In this study, the values of *F*, *N*_*f*_, *N*, and *R*_0_ on both host plants are completely consistent with the proven relationship. This relationship can be used to detect errors in life table analysis.

Although the weevil could survive longer than 160 days on both host plants (Fig. 1), the reproductive values (Fig. 4) showed that female adults did not contribute to the population growth after 92 d when reared on *I. batatas*, while *C. formicarius* on *I. triloba* did not have any contribution to population growth after 116 d. It seems that females from *I. triloba* can migrate to nearby *I. batatas* field after 92 d and still produce offspring.

Many plants function as oviposition sites for most herbivorous insects which deposit their eggs on all parts of the plants^31^. Leaf boundary layer effects both insect egg deposition behavior and progress of the embryo inside the egg^31^,^32^. The effects of eggs on plants consist of egg-induced changes of photosynthetic activity and of the plant’s secondary metabolism^31^. In this study, out of 150 eggs, four eggs did not hatched. This could be possibly due to ovicidal effect of host plants of *I. batatas* or *I. triloba*^32^,^33^.

A population projection based on an age-stage, two-sex life table can reveal the change of stage structure during population growth. Understanding stage structure is important to pest management because the dispersal and damage capability of insects varies with stage. This study demonstrates that such a life table can provide a comprehensive description of the fitness of an insect population on a given host plant. This study provides an interesting information on life table comparison of a *C. formicarius* on two of its host species. This shows that implications of the life-table data in IPM and/or weevil biology. The measure of the degree of dispersal from morning glory to sweet potato is clear, therefore the morning glory management around sweet potato fields is warranted. However, further studies are required on some insects occurring in alternate host plants may have to be proven to belong to genetically distinct populations or even cryptic species that do not cause much damage to crops of interest. Additional studies are also required if *I. triloba* is also the alternative host of another pest of sweet potato (i.e., *Euscepes postfasciatus*) an IPM strategy to manage *I. triloba* needs to consider *E. postfasciatus* in the picture.

This study determined that the *C. formicarius* can survive, on average, longer than four months on both host plants, and successfully produce offspring for a month. Because *I. triloba* can be used successfully by *C. formicarius* as an alternate host, we suggest that growers should include the clearing of *I. triloba* from around sweet potato fields and storage facilities in their management program.

## Additional Information

**How to cite this article**: Reddy, G. V. P. and Chi, H. Demographic comparison of sweetpotato weevil reared on a major host, *Ipomoea batatas*, and an alternative host, *I. triloba*. *Sci. Rep.* **5**, 11871; doi: 10.1038/srep11871 (2015).

## Figures and Tables

**Table 1:** Means and standard errors of developmental time (d), adult longevity (d), and fecundity (eggs), adult preoviposition period (APOP) (d), total preoviposition period (TPOP) (d) of *Cylas formicarius* (F.) reared on *Ipomoea batatas* and *Ipomoea triloba.*

Host plant					
Stage	*n*	*I. batatas*	*n*	*I. triloba*	*P*
Egg	149	4.86 ± 0.25	150	5.93 ± 0.15	0.0000
Larva	146	37.08 ± 0.22	150	54.87 ± 0.54	0.0000
Pupa	146	8.32 ± 0.15	150	16.99 ± 0.29	0.0000
Female adult	90	87.87 ± 1.11	83	57.41 ± 0.95	0.0000
Male adult	56	88.77 ± 1.32	67	56.25 ± 1.47	0.0000
APOP (day)	90	0.10 ± 0.03	83	0.07 ± 0.03	0.5243
TPOP (day)	90	50.16 ± 0.39	83	77.16 ± 0.88	0.0000
Oviposition days	90	29.72 ± 0.19	83	22.80 ± 0.17	0.0000
Fecundity (eggs)	90	90.91 ± 0.98	83	68.57 ± 1.24	0.0000

The difference between two treatments were evaluated by using paired bootstrap test.

**Table 2:** Means and standard errors of the intrinsic rate of increase (*r*), finite rate (λ), net reproductive rate (*R*_0_), and mean generation time (*T*) of *Cylas formicarius* (F.) reared on *Ipomoea batatas* and *Ipomoea triloba* estimated by using bootstrap technique with 40,000 resamplings.

Host plant			
Parameters	*I. batatas*	*I. triloba*	*P*
*r* (d^−1^)	0.0642 ± 0.0012	0.0418 ± 0.0010	0.0000
*λ* (d^−1^)	1.0663 ± 0.0013	1.0426 ± 0.0010	0.0000
*R*_0_ (offspring/individual)	54.55 ± 3.70	37.94 ± 2.89	0.0004
*T* (d)	62.26 ± 0.44	87.07 ± 0.92	0.0000

The data of two treatments were compared by using paired bootstrap test.
